# The system of autono‑mobility: computer vision and urban complexity—reflections on artificial intelligence at urban scale

**DOI:** 10.1007/s00146-022-01590-0

**Published:** 2023-05-08

**Authors:** Fabio Iapaolo

**Affiliations:** Faculty of Technology, Design and Environment, Oxford Brookes University, Oxford, UK

**Keywords:** Self-driving cars, Computer vision, Urban AI, Autono-mobility, Machine learning, Autonomous technologies, Urban governance

## Abstract

Focused on city-scale automation, and using self-driving cars (SDCs) as a case study, this article reflects on the role of AI—and in particular, computer vision systems used for mapping and navigation—as a catalyst for urban transformation. Urban research commonly presents AI and cities as having a one-way cause-and-effect relationship, giving undue weight to AI’s impact on cities and overlooking the role of cities in shaping AI. Working at the intersection of data science and social research, this paper aims to counter this trend by exploring the reverse perspective: how do cities affect the development, and expose the present limits, of SDCs? The contribution of this paper is threefold. First, by comparing urban and nonurban environments and thoroughly examining the relationship between computer vision and city-specific sociality and form, it defines machine autonomy/automation as a function of the sociotechnical milieu in which an AI system operates. Second, and related, the paper problematizes the notion of SDCs as autonomous technologies and the role it plays in envisioning contending policy arrangements and technical solutions for achieving full driving automation. Finally, the article offers insight into a materialist and spatialized understanding of AI—namely, not as an abstract quality susceptible to replication within discrete machines, but rather as a distributed property emerging through embodied interactions among a multiplicity of agents (human, non-human, and technological) within/with their environments.

## Introduction

At the time of its first appearance on American public roads at the beginning of the twentieth century, the automobile was considered a luxury item reserved for the exclusive use and delight of a small number of wealthy enthusiasts and practitioners (Norton 2008). Since about the 1960s, the car has become ubiquitous (Dant 2004), establishing itself not just as an undisputed symbol of modernity (Lefebvre 1971), but also as one of the main catalysts for urban transformation—one capable of reshaping urban geography in its own image. Notoriously, for the automobile to become a mass medium of urban transportation (Dant and Martin 2001), profound cultural, regulatory, economic, and infrastructural changes were necessary over the past century (Featherstone et al. 2005). Cities, in other words, had to be rearranged, socially and spatially, around cars (Norton 2008). For decades now, automobility has been considered “a common feature of everyday life itself, almost a background to the background” (Thrift 2004, pp. 45–46). However, the recent surge of interest in AI technologies has brought automobility back into the forefront of discussions about urban governance and planning. Such renewed attention is due primarily to the potential commercialization, in the near or distant future, of self-driving cars (SDCs)—namely, vehicles capable of automating all those functions that in traditional cars are governed by a human driver, including moment-to-moment decisions within high-stake, morally ambiguous situations. No longer perceived as futuristic objects from science fiction, SDCs are now a reality. Presently, positions about their possible introduction into urban roads are split. While some consider it inevitable (Claudel and Ratti 2015), others regard it as fantasy deemed to remain so, at least for a few decades (Casner et al. 2016; Janai et al. 2020). Either way, there is a general agreement on the great potential of SDCs to radically reshape, as the motorcar did throughout the twentieth century, multiple aspects of urban life. How this will happen is currently subject to much debate and speculation among urban planners and engineers, architects, jurists, ethicists, and city governments.

For their advocates, including automakers, software providers, and institutional actors, SDCs have the potential to yield significant social gains, such as enhanced safety, accessibility, efficiency, and sustainability. Increased road safety, in particular, provides the primary justification for their political acceptability. According to oft-reported estimates, around 94% of all fatal crashes are due to human error. By removing human error from the driving equation, SDCs promise a drastic reduction in road accidents and fatalities. No longer requiring a person behind the wheel, SDCs are also believed to allow for more personal leisure, while expanding access to point-to-point mobility to previously underserved populations, such as the elderly and visually impaired (Woyke 2016). Another expected benefit is that, combined with emerging trends in car sharing, SDCs could ease traffic congestion with a projected 80% reduction in privately owned vehicles (Claudel and Ratti 2015). As a result, scarce urban assets like land and buildings used for parking could be reallocated for other purposes (Ratti and Biderman 2017).

Notwithstanding the potential benefits listed above, there are still many unanswered questions regarding their short- and medium-term effects, including how they will disrupt and reshape cities’ political economy, public infrastructure, people’s experience of space, and urban design itself. In this regard, it suffices to think of the potential cascading effects resulting from the marginalization or displacement of taxi drivers and other jobs currently requiring a human behind the wheel (Maughan 2019). Further eluding anticipation are the potential negative effects on broader political and socio-economic arrangements. The significant investments required for the city-wide deployment of SDCs will likely divert resources away from public transportation and other crucial policy domains, such as healthcare and education (Blyth et al. 2016). Widely debated are issues of liability and accountability in the event of accidents (Ganesh 2017). There is currently a regulatory gap concerning international safety and legal standards that makes it unclear who would be held legally responsible for any injuries or property damage caused by the vehicle, such as the owner, manufacturer, or code developers (Schellekens 2015). Additionally, the extensive use of software in SDCs raises concerns about the potential risks of hacking attacks (Maughan 2019).

About one century ago, the automobile (or ‘horseless carriage’ as it was also referred to at the time) promised to modernize a personal mobility system primarily reliant on animal-pulled vehicles, which car advocates considered ill-suited to modern society’s needs (Norton 2008; Tarr and Mcshane 2007). However, the extensive, far-reaching impacts, both intended and unintended (such as urban sprawl and air pollution), of the automobile were not fully understood until much later (Jacobs 1961). Currently, claims in favor of adopting SDCs from public and private actors, such as Waymo, Tesla, Uber, and others, reflect a vision for the future that seems both politically desirable and technologically inevitable. From their perspective, the transition from manual to fully automated vehicles is viewed primarily as a technological advancement publicly justified based on the expectation that SDCs will remedy the negative externalities associated with traditional cars.^1^

This vision, however, is grounded upon the flawed assumption that the transition to full driving automation will be straightforward and without political tensions. SDCs are often marketed as a technology that will significantly ameliorate city life while maintaining existing socio-spatial systems substantially unchanged. However, this is unlikely to be the case. The mundane truth is that, as concisely yet powerfully stated by Stilgoe (2017b, p. 5), “[t]his plug-and-play story, in which the car is seen as able to get along with the world’s complexities as they are, without making additional demands, is a lie”. In other words, due to manifest incompatibilities between the technology’s operating logics and current urban infrastructure and social behaviors, significant changes will be required before SDCs can be used in cities. Specifically, it is argued here, cities will be reshaped, socially and spatially, to accommodate the preemptive logics of machine vision systems used to map the vehicle’s surroundings and predict future occurrences happening therein.

Focused on city-scale automation and using SDCs as a case study, this article reflects on the role of AI, and in particular computer vision—the main technical issue addressed and one of considerable practical policy and engineering import—as a catalyst for urban transformation.^2^ So far, critiques of SDCs have mainly centered around ethical and legal ambiguities due to their autonomous operations and decision-making (Ganesh 2017); the contested governance of social innovation (Blyth et al. 2016; Stilgoe 2017a, 2017b; Taeihagh and Lim 2019; Marres 2020); the uneven distribution of power within complex human-technical assemblages (Bissell 2018; Ganesh 2020); the erosion of cognitive skills exteriorized into machines (Casner et al. 2020); and their broader societal impacts (Bissell et al. 2020). Only recently, scholars have started to examine the specific urban implications of SDCs (Duarte and Ratti 2018). Notwithstanding, apart from a few exceptions (Stilgoe 2017b; Bissell 2018; Iapaolo 2019; Bissell et al. 2020), urban research commonly presents SDCs and cities as having a one-way cause-and-effect relationship, putting excessive focus on AI's effect on cities and neglecting the impact of cities on AI. This paper challenges the prevailing techno-deterministic views by exploring the reverse perspective: how do cities affect the development—and expose the present limits—of SDCs?^3^

An SDC can be thought of as either a purely technical assemblage consisting of hardware and software that function together in executing the driving task (e.g., sensorimotor systems, computer vision, and machine learning algorithms), or as a sociotechnical assemblage embedded “within larger interlocking systems, rather than … as [a] discrete entit[y]” (Bissell et al. 2020, p.10). An SDC, in other words, can be seen as part of what I define here, rephrasing what Urry (2005) wrote about traditional cars almost two decades ago, as a *system of autono-mobility*, which encompasses more than just vehicles but also includes physical and digital infrastructure, machine learning algorithms, training datasets, geolocation and mapping systems, three-dimensional cartographies, laws and codes of the road, roboethics, mobility cultures, governance structures, and new social habits and lifestyles.

The methodology of this article, which aims to bridge the gap between social research and computer science by combining advances from both fields, is based on a triangular emphasis that dialogically explores the political, spatial, and technical aspects of AI (see Hayles 1990).^4^ Following Pasquinelli’s (2019, p. 3, italic in the original) admonishment that existing research and debates on AI (see Ouchchy 2020) frequently “remai[n] at the level of speculation (‘*what if AI’*) and fai[l] at clarifying machine learning inner logic and intrinsic limits (‘*what is AI*’)”, this paper explores the underlying computational logics of SDCs in relation to city-specific sociality and form. Importantly, it does so by reintroducing materiality alongside technicality as key aspects for a better appreciation of the spatialized effects of AI.

Theoretical in scope and interdisciplinary in orientation, the contribution of this paper is threefold. First, by comparing urban and nonurban environments, it defines machine autonomy/automation as a function of the sociotechnical milieu in which an AI system operates. Here, I endorse Kitchin’s (2017, see also Hayles 2017) approach to researching algorithms, which posits that the focus should not be on algorithms alone but rather on the broader sociotechnical assemblages they are part of, including “social practices, material properties, discourses, mathematical abstractions, and code” (Matzner 2019, p. 4). This perspective recognizes that automated actions and decisions are shaped by the intricate interplay between algorithms and the technical elements and subsystems they are networked with (e.g., sensorimotor subsystems). At the same time, they depend on contextual factors, arising through reciprocal interactions with the infrastructure of the built environment and the physical world of objects and people (Kitchin and Dodge 2011, see also Blanchette 2012). Drawing on the state-of-the-art in driving research, the article then delves into some of the key challenges currently hindering the introduction of SDCs into cities. By exposing how machine vision systems operate—and fail—within environments designed for the human senses, the paper problematizes the notion of SDCs as autonomous technologies and its role in envisioning contending policy arrangements and practical solutions for achieving full driving automation. Proposing the conceptual lens of *autono-mobility*, it is argued that a precondition for the city-wide deployment of SDCs will be the social and spatial reconfiguration of urban spaces to compensate for today’s weaknesses of machine learning and vision—with requirements (social, political, spatial, and environmental) extending within and beyond the immediate urban surroundings. In the conclusive remarks, the article makes the argument for a materialist and spatialized understanding of AI—namely, not as an abstract quality susceptible to replication within discrete machines, but rather as a distributed property emerging through embodied interactions among a multiplicity of agents (human, non-human, and technological) within/with their sociotechnical environments.

## Artificial sensorium

In the last few years, SDCs have been trending in discussions of the future of mobility, “captur[ing] the popular imagination arguably more so than any other transportation technology over the past half century” (Bissell et al. 2020, p. 117). Nevertheless, or perhaps precisely because of that, an important aspect entirely overlooked in the current debates about self-driving technology is that automated transport systems are already fully operational in nonurban settings. Examples include automatic shuttles moving people between and within airport terminals or unmanned vehicles used for good transportation in industrial settings, such as factories, mines, and ports (see Chu et al. 2018, on the future of automated ports). These vehicles embody a degree of technological sophistication much less advanced than that SDCs do. Yet, unlike the latter, they are employed within standardized, controlled, and highly predictable environments, where the range of unexpected situations and human-related events the vehicle has to handle is very limited. An automatic train, for example, operates on a predetermined path with set stops, making its functions—and the task environment—predictable and thereby pre-programmable. Similarly, driverless vehicles utilized in industrial settings automate tasks in standardized and single-functional spaces with minimal-to-no interaction with other vehicles or people.

On the other hand, SDCs are designed to work in complex, non-predictable, and information-rich environments, which is exactly what urban roads are. On a busy road, an SDC must react within milliseconds to a wide range of situations that cannot be fully anticipated. City streets, due to their high density, diverse architecture, and variety of road users, add even more uncertainty to the environment. Traffic coordination can be seen as a classic instance of joint action (Chater et al. 2018), with mutual strangers negotiating intended actions and decisions according to shared rules. In dense city traffic, a car must simultaneously interact with a multitude of actors (e.g., pedestrians, bike runners, other vehicles, and animals), each acting independently in complicated ways. At any given moment, an SDC must, to mention but a few examples, continuously adapt to changing circumstances such as traffic speed; observe traffic rules; handle ambiguous situations (e.g., hand signals from traffic officers or construction workers); interpret unwritten conventions, and react to emergencies or rule-breaking behavior by other road participants (e.g., a pedestrian crossing the street in a non-designated section of the roadway).

Due to the high degree of uncertainty in their task environment and, consequently, the high unpredictability inherent to their operations, it has become standard practice to refer to SDCs as autonomous technologies. That is because the way SDCs operate involves a wide *degree of indeterminacy*,^5^ here understood as the extent to which the vehicle’s behavior is continuously adaptive to contingent factors and situations, making it difficult to be governed through formal rules. SDCs operate in a way that is predictable at a high level (e.g., the vehicle will go from point A to point B), but not moment by moment, leading to the possibility of unexpected outcomes. To ensure responsiveness to external stimuli, SDCs use machine learning algorithms, sensorimotor technologies, and geo-referenced mapping and positioning systems. In concert, these technologies embody complex forms of cognition (Hayles 2017) that allow the vehicle to perceive and understand its surroundings and choose the best possible course of action from various alternatives. In this sense—and in this sense only—an SDC can be said to be operating *autonomously* from its designers and end-users.

Compared to traditional, logic-based forms of automation where outcomes are “already implicated in initial premises” (Parisi 2019, p. 3), SDCs have become paradigmatic of widespread conceptualizations of AI as discrete technologies or technical systems, the most advanced of which are said to be capable of “operat[ing] without the need for human intervention or supervision, mak[ing] decisions independently, and accommodat[ing] to changed circumstances” (Kaplan 2016, p. 147). Although it may be misleading, the common practice of extending autonomy beyond the human to include technology provides valuable insights. In this context, the term ‘autonomy’ encompasses both a political and technical meaning. Politically, it symbolizes the popular imagination, or rather misconception, of SDCs as “neoliberal, individualised agent[s] … that can act independently and efficiently on the basis of guidelines and feedback” (Ganesh 2017, p. 7). Technically, it speaks for the advancement of sensing and cognitive abilities in current and developing AI technologies (see Lynch and Del Casino 2020; Hayles 2017).

Despite business claims (see Tennant and Stilgoe 2021) and popular ideas about technology’s autonomy (see Winner 1977), SDCs do not exist in a vacuum. They are quintessentially socio-spatial agents whose main goal is to transport passengers from one point to another. In the process, they navigate a world shared with many other entities (see Mattern 2017): humans (pedestrians, cyclists), animals, objects (road signs, street furniture), and whatever elements they encounter during their journey. To safely perform their task and avoid collisions, SDCs must be able to sense and interpret their surroundings under any weather and lighting conditions. In a sense, SDCs instantiate what the French philosopher and urbanist Paul Virilio (1994, p. 59), writing in the Eighties, defined as *sightless vision*, in which “the capacity to analyse the ambient environment and automatically interpret the meaning of events” is delegated to the dyad computer–camera. In driving automation, that which with anthropomorphic vocabulary is named machine perception, in reality, refers to complex statistical and probabilistic models used to identify the type and location of nearby objects and anticipate their behavior in the near future. Manovich (1996, p. 12) observes that “in the field of computer vision, “understanding a scene” implies two goals. First, it means the identification of various objects represented in an image. Second, it means reconstruction of three-dimensional space from image”.^6^ In SDCs, machine perception relies on four different types of sensors: cameras, radars, ultrasound sensors, and LiDAR scanners. Schematically, the overall decisional loop of an SDC follows a perception-decision-action sequence. During the perception stage, sensor readings are used to create a real-time spatiotemporal view of the vehicle itself in relation to its surroundings, including other vehicles, road signs, and street elements. Combined with prior knowledge of the road infrastructure and driving rules, this information is then used to calculate driving decisions that are executed by the vehicle's mechanical actuators.

It is important to note that exteroception (the perception of the road environment) is never 100% accurate. Just like human decision-making, decisions made by SDCs are always based on incomplete and imperfect information. Sources of uncertainty can come from either internal elements within the vehicle, such as faulty sensors or data processing mistakes, or external factors. To mitigate internal uncertainty, SDCs are equipped with multiple sensors of different types. The rationale is that fusing data from multiple sensor sources helps achieve more accurate results compared to relying on just one (Hall and Llinas 1997). However, in complex urban environments, the largest source of uncertainty is usually external and related to real-time coordination with other traffic participants and the road infrastructure.

Up until today, urban roads have been built around people and, with the rapid rise of automotive traffic, around motorists primarily. By “impos[ing] a strong social control over the most fundamental of human behaviors, whether to move or be still” (McShane 1999, p. 370), traffic signing systems (i.e., traffic lights, road signs, and painted pavement) are one crucial element of the road infrastructure. Used to convey helpful information for navigation—restrictions, prohibitions, and warnings—they dictate behavior and facilitate coordination among traffic participants. International design principles for traffic signing systems are regulated under the Vienna Convention on Signs and Signals (1968). The convention categorizes road signs into seven classes, such as danger warning and priority signs, and specifies the color, size, and shape of each class. It also outlines the requirements for road markings, such as length, width, color, material, and message, as well as the colors and meanings of traffic lights.

Understandably, these standards have been defined to meet the demands of the human eye; that is, to minimize the time gap between the presentation of the stimulus and the driver's enaction of the appropriate response. Road signs, for instance, are strategically positioned for easy and unambiguous interpretation by drivers, particularly while driving at high speeds. By the same logic, road markings use light-colored retroreflective materials for maximum visibility during both day and night. During the day, they are visible due to their contrast against the dark pavement, while at night, retroreflective pigments bounce light back from the vehicle’s headlights. Since the visual stimuli that mediate urban flows are designed for human and not machine vision, interpreting traffic signs and reacting to signaling are challenging tasks for SDCs, especially in busy city environments where significant computational resources are required to detect and track moving objects.

A partial solution to problem, one which, following Waymo (2016), is now common to most manufacturers, is the combination of real-time sensor data with centimeter-accurate maps that provide contextual knowledge on, for example, lane geometries, traffic rules, and location of traffic lights. This approach not only facilitates the vehicle's self-location in the absence of GPS, but also reduces the computational load otherwise needed for mapping permanent features of the built environment. Urban roads are, however, fast-changing environments (e.g., due to new infrastructure or regulations). Admittedly (see Waymo 2021), building, maintaining, and updating maps with such fine detail is a prohibitively costly—labor-intensive and time-consuming—activity. The process entails the use of human-operated vehicles equipped with LiDAR technology to capture 3D images of selected urban areas. These images must then undergo a meticulous manual review and validation before they can be utilized by SDCs.

The political significance of such an impressive cartographic project is easily recognizable (see Hardigree in 2019). Since SDCs are only capable of navigating pre-mapped and geo-fenced roads, these maps essentially define the geographical scope of the technology, limiting its adoption to those cities or urban areas deemed investment-worthy. Cartography, as highlighted by Denis Wood in his seminal book *The Power of Maps* (1992), is not just a scientific discipline but also a political endeavor aimed at securing mapmakers’ control over territories. The high-resolution maps used for SDCs are not neutral either, but rather reflect the interests of their creators. This raises various questions, such as who has the authority to decide which areas should be mapped and made accessible, who sets the priorities, and how the process of making urban roads intelligible to SDCs will either reinforce existing or create new socio-economic divides within and between cities.

In addition to GPS signal disruption or loss due to tunnels or *urban canyons* (Cui and Ge 2003),^7^ two classic problems for computer vision in urban settings are road sign blockages caused by trees or other occasional occluding elements (Muoio 2016) and reduced sensor performance in adverse lighting and weather conditions (Zang et al. 2019). The limitations of cameras and LiDAR technology in poor weather conditions, such as fog, heavy rain, or snowfall, are well documented (Dannheim et al. 2014). Additionally, minor issues that would not significantly affect human vision, such as faded or leaf-covered road markings, can cause problematic outcomes like off-course driving or unnecessary stops (Sage 2016; Flockett 2017; Barut 2018). Sporadic manifestations of latent flaws in computer vision include mistaking mannequins for pedestrians (Waymo 2021) or the moon for a yellow traffic light (Ramey 2021). Although some of these errors can be harmless, hilarious even, such as Tesla's autopilot confusing a Burger King sign for a stop sign (Lambert 2020), they can also pose a danger in safety–critical situations, as demonstrated by the fatal 2016 Tesla Model S accident (Yadron and Tynan 2016).

Just like any other technology, computer vision systems are susceptible to both deliberate and unintentional *adversarial attacks*. As shown in a study by Eykholt et al. (2018), even minor modifications to road signs, such as graffiti or stickers (Field 2017), can deceive computer vision systems. The researchers demonstrated that modest changes to a stop sign, like altering the background or adding graffiti, can result in the system mistaking it for a 45-mile-an-hour speed limit sign, causing the vehicle to speed up instead of stopping. In another recent experiment conducted within a controlled setting (Tencent Keen Security Lab 2019), stickers placed on the road prompted a Tesla Model S to switch lanes and drive abruptly into oncoming traffic. These studies highlight the vulnerability of current computer vision systems to even simple forms of deception. If such incidents were to occur in real-world scenarios, the consequences could be serious.

In densely populated urban areas, the interaction between SDCs and vulnerable road users, especially pedestrians and cyclists, can be particularly problematic. This is due to two main reasons. First, the unpredictable movements and changing directions of pedestrians and cyclists make it difficult to predict their behavioral patterns. Second, people’s appearance variability (e.g., physical attributes or clothing) negatively affects algorithms used for human detection and classification (Janai et al. 2020). These algorithms rely on supervised learning, where they are taught to recognize specific categories, such as differentiating between pedestrians and cyclists, through labeled examples. Indeed, before they can successfully identify anything, algorithms must first be instructed on what to see in the first place. However, in real-world scenarios, human unpredictability and variability limit their ability to apply their training effectively.

Also, in real-world situations, unexpected phenomena can emerge “that the training data simply did not include and could not have anticipated” (Gillespie 2014). The widespread use of masks during the COVID-19 pandemic highlights this issue. Commercial facial recognition systems like Apple Face ID, which had been trained on pre-pandemic images, suddenly had difficulties recognizing masked individuals (Simonite 2020). To address this problem, new training datasets were required, and in some cases, this was achieved by overlaying computer-generated masks on existing facial datasets (Ngan et al. 2020). Given that novel situations simply cannot be anticipated, it is likely that SDCs will face problems in the future comparable to those experienced by commercial software technology today—with even higher stakes involved.

These examples demonstrate that computer vision relies not only on algorithms and sensors, but also, and perhaps more significantly, on training datasets. Training datasets play a vital role in determining what SDCs can *see* and how they *behave*, even more so than the algorithms that govern the vehicle’s behavior in real time (see Beer 2017 on the social power of algorithms). In driving automation, the quality of training datasets depends on various factors, such as the selection of useful taxonomies, categories, and subcategories; accuracy in data preparation and labeling; availability of training and test datasets reflecting all potential scenarios self-driving cars may face in real-world conditions, including *corner cases* (e.g., cars disregarding traffic signals)^8^; and the method of data collection, such as open-source datasets like ImageNet or ground truth data collected under actual traffic conditions (e.g., the KITTI dataset).^9^

These elements form the foundation of computer vision. They can be viewed as components in a machine learning assembly line (Pasquinelli and Joler 2020), the layered intricacies of which often conceal the fact that human decisions always already determine the extent and scope of machine vision. Far from merely defining the technological capabilities and limitations of computer vision, training datasets are, more evidently so than algorithms, imbued with values, assumptions, worldviews, or, to say it in one word, *politics*—in the sense of Winner (1980). Crawford and Paglen (2019) observe that "the automated interpretation of images is an inherently social and political project, rather than a purely technical one". Indeed, computer vision implies much more than just receiving and processing environmental cues; it also involves valuing and prioritizing certain things based on cultural norms and expectations. In this regard, if there is any important lesson to be learned from the famous "Trolley Problem" is that, contrary to its intended purpose (Awad et al. 2018, p. 59), there cannot be easily determined "global, socially acceptable principles" to guide engineers and programmers as values vary among cultures and circumstances (see Gold et al. 2014).

The central, and always open to negotiation (Amoore 2020), political challenge becomes determining what should be prioritized and made visible and what should be overlooked and invisibilised. In driving automation, nowhere is this more evident than in the initial specification of targets of interest (e.g., pedestrian, cyclist, or vehicle), though other factors also play a crucial role. For instance, SDCs have already been shown to have difficulty recognizing people with darker skin tones (Ganesh 2020, see Wilson et al. 2019). An unwanted consequence due to the uneven representation of different ethnic and social groups in the training data, this situation still illustrates the much-debated problem of social biases being amplified as they enter the realm of computation—in this case, by rendering minorities even less visible through the automation of perception tasks.

Until today, car manufacturers have primarily focused on upgrading their vehicles' technology to address the challenges of SDCs and mitigate the uncertainties that come with them. This has often involved equipping the cars with a more significant number and variety of sensors to ensure data redundancy but with the trade-off of greater computational costs and longer execution times. Nevertheless, as we will see later, it is becoming increasingly clear that vehicle-based solutions alone are not sufficient. The widespread integration of SDCs into cities will require not only technical advancements in areas such as machine learning and computer vision, but also a social and material restructuring of urban areas to create a supportive environment for SDCs.

## The system of autono-mobility

State-of-the-art self-driving technology is not yet ready to handle urban complexity. Arguably, computer vision’s unreliability in crowded city traffic is currently the main barrier to adopting SDCs in urban areas. Despite this, in the wake of what some have termed “testbed urbanism” (Halpern et al. 2013, see Kitchin 2014), in various cities around the world, there has been a push for street trials conducted by car manufacturers in partnership with local governments and other relevant stakeholders like software providers, universities, and private research centers. Famous examples include Waymo's partnership with the state of California (Subin and Wayland 2021) and Uber's partnership with Arizona, later temporarily suspended after a fatal pedestrian accident in 2018 (Bradshaw 2018).

Policies and legislation regarding SDCs vary from country to country and city to city, reflecting different, and sometimes conflicting, perspectives on driving automation. For example, in the United States, driving automation is primarily seen as the capability of individual vehicles to operate autonomously. This car-centered viewpoint holds that private technology companies, not government efforts, will drive the transition toward full driving automation by creating the required technology. In Europe, conversely, "the debate on connected cars still prevails over autonomous vehicles, feasibility of which is often questioned by EU officials under European driving conditions with complicated city centers" (FTI Consulting 2017, p. 2). The underlying idea is to develop cooperative/intelligent transport systems where vehicles are connected to each other and infrastructure through wireless technologies such as vehicle-to-vehicle and vehicle-to-everything communications (see Ionita 2017).^10^

Despite these variations, some trends are ubiquitous. In the absence of an international agreement on the regulation of SDCs, local governments are implementing regulations locally. To promote mutual benefits, so far, they have restricted their role to granting authorization, offering incentives, and potentially suspending on-road trials, while allowing major market players to take the lead in the research and development of SDCs. This "wait-and-see" approach (Grieman 2019) demonstrates confidence in the potential benefits of SDCs, such as improved safety and efficiency, but also assumes that the move toward full driving automation will not require significant public investment. On the other hand, for car manufacturers, gaining access to public roads can provide a significant competitive advantage and lead to lock-in effects, as the data gathered from real-life traffic conditions is proprietary.

Due to a number of accidents involving semi-automated or fully automated vehicle prototypes, in recent years, car manufacturers have faced criticism for prioritizing their interests over public safety. More recently, however, there has been a shift in the public discourse, with increasing concerns being voiced by car manufacturers themselves and other key private players (see Stilgoe 2017a). Among these, one commonly held view is that the main factor presently hindering the widespread adoption of SDCs in cities is a lack of government funding for the necessary infrastructure. As a result, public scrutiny has shifted from questioning the capability of SDCs to safely navigate existing urban roads to problematizing cities for being ill-suited to the technology's requirements. The underlying idea is that, while there may still be some room for further advancement, self-driving technology has reached maturity. For Claudel and Ratti (2015), for instance, "[f]rom a technological point of view, driverless cars have arrived; the bigger task is for cities to integrate them". Likewise, Duval et al. (2019) argue that "[w]ithin cities, at least, a fully autonomous world awaits. Even though this world may be many years down the road, public officials should understand the changes ahead and consider the modifications needed to accommodate such systems". On the same lines, Oliver et al. (2018) maintain that "the key question we should be asking is not when will self-driving cars be ready for the roads, but rather which roads will be ready for self-driving cars". This almost unanimous call for greater involvement from public actors in the development and regulation of SDCs has two consequences. First, it redefines the political significance of those involved in governing and developing SDCs. Second, it lays the foundations for new practical solutions and policy arrangements for implementing SDCs in the short and long term.

As noted earlier, up until now, car manufacturers have primarily concentrated on enhancing the technology within their vehicles to cope with the challenges posed by urban areas. However, a more effective approach could be to alter the external environment to make it less complex for vehicles to navigate. This would entail transforming the urban landscape, including its design, material components, and social aspects, to overcome the limitations in computer vision technology. There are various ways to accomplish this, ranging from minor modifications to existing infrastructure to more ambitious plans for creating ‘smart roads’ that would optimize traffic flow through the use of wireless communication between vehicles and the road environment (Duvall et al. 2019). Ford, for instance, is testing a similar approach in Miami-Dade County, Florida. At critical intersections, the company has installed so-called ‘smart nodes’ equipped with cameras, radar, and LiDAR sensors. These nodes provide supplementary information to the data collected by the car's onboard sensors, such as other vehicles approaching a traffic light (Ford Motor Company 2021).

However, in a world where many municipalities struggle to maintain even ordinary roadways, the development of advanced smart infrastructure seems to be a distant goal for the time being. In the meantime, traditional vehicles and SDCs will likely coexist, yet with measures aimed at preventing or controlling their interactions. Lately, the idea of separating SDCs from other road users has gained traction, with various trials underway globally (Mullin 2020). This would involve creating dedicated lanes for SDCs with physical barriers separating them from human-operated vehicles (Krisher and Eggert 2020). Another solution could be limiting their use to slow-speed areas or structured environments such as urban highways (Hawkins 2018).

These approaches are more practical and economically viable compared to the extensive infrastructure changes needed in the long run. Nonetheless, the simplification of cities for SDCs will have an impact on both the built infrastructure and the social fabric of cities, potentially resulting in new forms of spatial segmentation and accompanying political conflicts. The history of the motor car highlights that urban roads have always been contested by many ‘street rivals’ competing for access to them (see Norton 2007). In the early 1900s, public streets were shared by various groups, including pedestrians, street vendors, children, and cyclists—all of whom had equal rights to the road. However, with the arrival of the motor car, cities witnessed the emergence of a new urban phenomenon, the car accident: "a new kind of mass death. Most of the dead were city people. Most of the car's urban victims were pedestrians, and most of the pedestrian victims were children and youths" (Norton 2008, p. 11). As cars, and their drivers, were initially seen, at best, as unruly intruders, city streets "had to be socially reconstructed as places where motorists unquestionably belonged" (Norton 2008, p. 1). Over time, the growing popularity of cars resulted in changes to city infrastructure, including the division of roads into specific areas for vehicles and others, more marginal, for pedestrians and cyclists. Along with these changes, new regulations were enacted to enforce these novel uses of the streets. In certain countries, including the United States, children were prohibited from playing on city streets, while the criminalization of 'jaywalking' restricted pedestrian freedom and rights. With the advent of SDCs, new forms of mobility and dwelling will emerge, but they will also require changes to current social practices and behaviors, including restrictions.

Urry (2004) pointed out that the widespread use of traditional cars as a personal mode of transportation was made possible by the concurrent growth of a globally expanding car system that gave them preference over other forms of mobility, such as walking, cycling, and rail. The author referred to this system as “automobility”—namely, “a self-organizing autopoietic, non-linear system that spreads world-wide, and includes cars, car-drivers, roads, petroleum supplies and many novel objects, technologies and signs” (Urry 2004, pp. 26–27). Similarly, the widespread adoption of SDCs will require its own supporting system, which I refer to here as *autono-mobility*. This system encompasses vehicles and other elements, such as physical and digital infrastructure, hardware and software technology, machine learning algorithms, intelligent traffic control systems, training datasets, machine-readable maps, changes in liability and insurance, and the evolution of social norms and practices.

It should be remarked that the scope of the said system extends within and beyond the urban scale. The ambitious project to introduce SDCs into urban roads is akin to a ‘planetary experiment’ (see Halpern 2019), with a wide range of potential implications not just for cities but political, economic, and spatial systems at multiple interrelated scales. As a result, it is crucial to conduct a comprehensive evaluation of SDCs that considers aspects often overlooked in discussions focused solely on cities.

At present, conversations about urban automation primarily center on the potential for AI to replace humans in driving and other domains. However, this narrow perspective disregards the disproportionate amounts of planetary resources required for that to occur. In driving automation, one such resource is the screen workers responsible for annotating images and videos used to train the computer vision systems of SDCs. These workers play a critical role in creating the data infrastructure that supports the computer vision systems in SDCs. Still, this infrastructure also comes with high environmental costs (see Sudhakar et al. 2023). Thus, while it is crucial to interrogate and possibly anticipate the impact of SDCs on cities, it is just as important to recognize the existing demands they already place on a global network of resources, labor, and data (Crawford and Joler 2018).

## Conclusion: on the spatiality and materiality of urban AI

This article has thoroughly examined the interplay between computer vision and city-specific sociality and spatiality. By taking a broader view of the sociotechnical system of autono-mobility, rather than just focusing on SDCs as the central unit of analysis, the article has made the case that urban transformation should not be solely viewed as a result of the integration of SDCs into cities, but rather as a necessary prerequisite for their successful deployment. Specifically, cities will need to undergo significant changes, socially and spatially, aimed accommodating the requirements and limitations of today's computer vision systems. Additionally, the study has advocated for a more comprehensive examination of the far-reaching, more-than-urban consequences of autono-mobility, including the planetary—social and environmental—costs implicated in the ongoing process of bringing fully automated vehicles onto city streets.

Yet, another important aspect needs to be emphasized: SDCs are nowhere near as autonomous as they are commonly portrayed. This is not to undermine their advanced cognitive abilities, as demonstrated by their capability to understand contextual information and adjust to changing surroundings (Hayles 2017). However, if applied uncritically to AI and SDCs specifically, the concept of autonomy is complicated for three main reasons.

First, in the near term, the most feasible approach to integrating SDCs into urban roads is limiting their functions by restricting their usage to more structured environments such as dedicated lanes. This approach would make the vehicle's behavior more predictable and, in a sense, less autonomous, similar to that of automated shuttles in airport complexes. Nonetheless, the idea of lowering the world’s complexities to increase technological autonomy is, at best, paradoxical. The same holds for long-term strategies. Currently, there is no consensus on the best way to manage the transition to full driving automation, except that it will require increased connectivity and interdependence between road actors to an unprecedented degree. In other words, enhancing road safety and improving traffic flow will be accomplished less through greater autonomy of individual vehicles and more through improving the interconnectivity among cars and between cars and road infrastructure, resulting in environments with collective cognitive capabilities that surpass those of individual actors.

Second, autonomy and intelligence are frequently mistaken as synonymous in discussions of AI, both among the general public and in academic circles, as if these traits are inherent in individuated technologies. This widespread perception of AI closely aligns with the liberal conception of humans as autonomous individuals possessing free will and innate intellectual faculties. However, this view disregards the extent to which intelligence is relational, embedded, situated, and infrastructural (Bruder 2019). Within an AI system, intelligence (and decision-making) can rarely, if ever, be attributed to one single entity. Instead, intelligent behavior is the outcome of complex interactions between multiple components and subsystems, each with its own material properties and limitations that influence the overall functioning of AI. At the same time, the situated decisions made by AI technologies, especially embodied systems like SDCs, are not made in isolation but shaped through mutual interactions with various human and non-human actors within a shared environment. Furthermore, as my comparative examination of urban and nonurban environments demonstrates, space is not just an incidental milieu but an integral aspect of AI that continuously shapes the affordances and limitations of the technologies it encompasses and interacts with.

Finally, it is essential to remember that AI is a human endeavor, encompassing all aspects from data collection to preparing training datasets and selecting the appropriate deployment environment. When humans disappear from the driving seat, it becomes ever more crucial to understand where else they are involved, including the relevant sites and scales where human decision-making continues to govern technology and affect its outcomes.

## Data Availability

Not applicable.

## Footnotes

1 The Society of Automotive Engineers International (2014) has proposed a general taxonomy for on-road motor vehicle classification, ranging from Level 0 (no automation) to Level 5 (full automation).

2 The expression AI is used here in a broad sense. To date, no consensus has emerged about what AI is (not least because the term *intelligence* itself eludes univocal conceptualization). Depending on one’s disciplinary perspective, analytical scale of interest (e.g., isolated algorithms or the broader sociotechnical systems of which they are but one element), and intellectual, political even, aims, AI can be understood in manifold ways. It is beyond my scope to provide a definition of AI. Yet, what needs to be emphasized is that AI is not just one single technology. Quite the opposite, the term is now commonly understood to be a general label for “a constellation of technologies” including, but not limited to, “machine learning, perception, reasoning, and natural language processing” (AI Institute 2016, p. 4).

3 As illustrated by Fuerth (2009, p. 20), the main problem with linearity as applied to the explanation of sociotechnical transformations is that it “distorts our notion of cause and effect. Under its influence, we tend to expect that for every problem there is a unique solution; and that proportionate changes of circumstances will produce proportionate changes of outputs. We believe that it is possible to disassemble (“unpack”) compound, conglomerate issues, without destroying their coherence”.

4 Technical knowledge on SDCs, and AI in general, has been gained through a systematic survey of refereed articles on the subject from engineer/computer science, college-level textbooks (e.g., Russel and Norvig, 2016), interviews with industry experts, as well as by visiting the research group: Artificial Intelligence and Media Philosophy (in German: *Künstliche Intelligenz und Medienphilosophie*, KIM), Karlsruhe University of Art and Design. For a technical survey on SDCs, see Yurtsever et al (2020). For a comprehensive overview on problems, datasets, and methods in computer vision for driving automation, see Janai et al (2020).

5 The expression *degree of indeterminacy* is borrowed from the French philosopher Gilbert Simondon (1958/2017, p. 17).

6 The author (Manovich 1996, p. 12) further explains that “[a] robot, for instance, needs not only to recognize particular objects, but it has to reconstruct a representation of the surrounding environment to plan its movements”.

7 That is, city streets flanked by high-rise buildings on both sides.

8 The test dataset comprises data (e.g., images) that the learning algorithm has never encountered before. For this reason, test data must be distinct from data used for training and validation.

9 Unlike other publicly available domain-generic datasets like ImageNet, the KITTI dataset is specifically meant for computer vision applications in automated driving. The KITTI dataset, which is becoming an important benchmark in the field, is based on discrete images retrieved from video sequences recorded around the mid-size city of Karlsruhe, Germany, using a human-piloted car equipped with various sensors, including cameras and LiDAR. See http://www.cvlibs.net/datasets/kitti/

10 One example being Barcelona ‘superblock’ initiative (Bausells 2017).
